# A hierarchical analysis of perceived team personality traits in sport

**DOI:** 10.3389/fspor.2025.1502988

**Published:** 2025-04-10

**Authors:** Chanho Kang, Jonathon Allen, Jim Watkins

**Affiliations:** Department of Kinesiology, University of North Alabama, Florence, AL, United States

**Keywords:** sport team personality, brand personality, lexical approach, the Big Five model of personality, HEXACO Honesty/Humility, sport brand management, hierarchical factor analysis

## Abstract

**Introduction:**

This study aims to revisit and enhance the foundational concept of perceived team personality by addressing critical conceptual and methodological challenges in previous brand personality studies. While prior studies have identified team personality dimensions and developed measurement scales, ongoing ambiguities in applying the general brand personality conceptualization to sport teams remain. In addition, the approach's limited generalizability, inadequate methods for selecting descriptors, and biases in team (brand) selection pose significant challenges to make a valid and reliable team personality scale in sports.

**Methods:**

To overcome these limitations, this study employs a lexical approach from personality psychology, which posits that fundamental personality dimensions emerge naturally from the adjectives people use to describe themselves and others. By analyzing a set of 99 sport-specific personality descriptors based on the lexical approach, this study explores hierarchical solutions ranging from one to six factors to determine whether perceived team personality dimensions align with established human personality models, such as the Big Five and HEXACO frameworks.

**Results:**

Findings reveal that the five- and six-factor models exhibit strong conceptual alignment with these established human personality structures, demonstrating the efficacy of the lexical approach in capturing sport team personality.

**Discussion:**

This research strengthens the theoretical and methodological foundation for assessing team personality in sport by providing a solid framework that better aligns with consumer perceptions. These insights may contribute to a more precise and contextually relevant understanding of team personality, offering implications for sport brand management, consumer engagement strategies, and long-term team positioning.

## Introduction

Team personality, operationally defined as consumers' perceptions of distinctive personality traits characterizing sport teams, has gained significant attention in sport brand research. Sport brand researchers have identified major team personality dimensions and developed valid and reliable scales to assess these key attributes in sport team brands (1–6). Previous research has found that these human characteristics attributed to brands play a significant role in differentiating them from competitors, enhancing brand loyalty and preference, and influencing purchase intentions and consumer behavior (7–11). Therefore, sport brand managers can strategically position and manage their teams by leveraging these scales to enhance brand image, strengthen consumer preferences, establish differentiation, and foster trust and long-term loyalty, even during poor team performance (4–6).

Despite the critical implications of team personality in sport, efforts to develop valid and reliable measurement scales remain limited (5). Additionally, conceptual ambiguities persist, making establishing a standardized framework for assessing team personality challenging (1–6). Similar to brand personality research in the broader field, there are two major approaches for conceptualizing and measuring team personality in sport (12–14). Studies following Aaker's (12) definition of brand personality conceptualize team personality as the set of human characteristics associated with a team (1–3, 5). By encompassing both personality traits and non-personality attributes based on Aaker's definition, the scales following the conceptual foundation can provide a broader and more comprehensive view of how consumers perceive sport brands (5, 12, 15).

Alternatively, studies influenced by Azoulay and Kapferer's (16) approach argue that team personality should only include human personality traits, excluding non-personality attributes related to situational, demographic, cultural, cognitive, or functional aspects rather than intrinsic psychological characteristics (4, 6, 13, 17). Both approaches acknowledge the limitations of directly applying Aaker's scale to measure sport brand or team personality while adopting either complementary or contrasting positions in methodology and conceptualization when developing scales to capture the distinct personalities of sport teams (4–6). Contrary to the cross-cultural generalizability of the Big Five and HEXACO models across different languages and cultures, previous studies have identified distinct sport brand (team) personality dimensions that do not align with Aaker's brand personality framework, the Big Five model, or the HEXACO model. These findings have significant implications for both research and practice in the field.

While influential, the direct adoption of Aaker's scale in sports contexts has faced substantial critique. This study addresses four major criticisms regarding (a) Aaker's (12) conceptualization of brand personality, (b) the limited applicability of Aaker's scale across product categories, (c) issues related to select descriptions of the construct, and (d) potential problems of brand selection. These concerns highlight the need for a more tailored and theoretically appropriate approach for sport team contexts.

To overcome these limitations, researchers may consider the lexical approach from personality psychology to address these issues. This approach posits that fundamental personality dimensions naturally emerge from adjectives commonly used by individuals to describe themselves and others (18–20). Extensively employed in personality psychology, this method provides a theoretically sound and contextually relevant strategy to identify key dimensions of team personality. Researchers can identify distinctive human personality traits relevant and applicable to sport teams by analyzing the language sport fans regularly use, ensuring alignment with fan perceptions of the teams they support.

Building on these insights, the present study addresses existing research gaps by employing the lexical approach to identify team personality dimensions. This study proposes that the lexical approach can offer a theoretically and methodologically solid framework for team personality analysis by examining sport consumer language. Furthermore, this study investigates whether these emergent dimensions align with established human personality frameworks, such as the Big Five and HEXACO models, to assess the lexical approach's applicability in understanding sport team personality.

## Review of literature

### Criticism regarding Aaker's conceptualization of brand personality

Aaker's (12) definition of brand personality—“the set of human characteristics associated with a brand”– has elicited criticism. This broad definition of brand personality may encompass other facets (e.g., physical facet) in the brand identity that is conceptually distinct from the brand personality concept (13, 16, 21). In personality psychology, personality refers to “relatively stable patterns of behavior, affect, and thinking” (22). Specifically, the term “personality trait” in psychology is considered to be a consistent or stable description of human personality over long periods of time. Therefore, brand personality can be conceptualized as consistent perceptions toward a brand instead of being caused externally, inconsistently, or reflective of temporary perceptions from consumers (16, 17).

Since personality traits should reflect enduring fundamental individual differences in tendencies of behaviors, thoughts, or feelings across a variety of relevant situations or over a relatively long period, brand personality traits should reflect an individual brand's enduring human characteristics that can be differentiated from other brands in the same product or service category. Given that terms such as “personality trait” or “human characteristics” have very specific meanings in psychology, Azoulay and Kapferer (16) argued that a new definition of brand personality should “remain close to that used in psychology, where the concept of personality has been analyzed for decades, although it should be adapted to brands” (p. 146).

In addition, Azoulay and Kapferer (16) argued that Aaker's definition of “brand personality encompasses dimensions conceptually distinct from the pure concept of personality” (p. 151). Based on the clarification of the concept of brand personality and its conceptual implication between personality in psychology and brand personality, Azoulay and Kapferer (16) defined brand personality as “the unique set of human personality traits both applicable and relevant to brands” (p. 153). Given this clarification of the brand personality concept, the current study utilizes the conceptual definition of brand personality by Azoulay and Kapferer (16).

Beyond these definitional distinctions, the assumption that brands inherently possess humanlike personality traits has been critically debated (14, 23, 24). While inanimate entities, such as non-human brands, can develop brand personality through marketing strategies, such as anthropomorphism, mascots, spokespersons, or anthropomorphic communication (25), previous research question whether consumers naturally perceive brands as humanlike entities (23, 24). Avis and his colleagues argue that if consumers do not inherently perceive brands as possessing human traits, the validity of attributing human personality characteristics to brands remains uncertain.

Although metaphorical thinking enables consumers to describe brands in human terms when explicitly prompted, this does not imply that brands inherently exhibit human characteristics or possess psychological personality traits (12, 23). Research has demonstrated that when directly asked, consumers can attribute human traits to inanimate objects, such as rocks (23). Avis and his colleagues argue that such perceptions may be constructed in research settings rather than occurring intuitively. Consequently, anthropomorphism may not be a default cognitive mechanism in brand perception, a notion often overlooked in brand personality studies.

Furthermore, neuroscientific evidence supports this distinction, as fMRI studies reveal different brain activation patterns when consumers think about brands vs. humans (26). While metaphorical thinking enables consumers to describe non-human entities in human personality terms when prompted, this does not necessarily reflect how brands are naturally processed in cognition (14, 23). Therefore, the tendency to attribute human personality traits to brands may be more reflective of situational framing and cognitive prompting rather than an inherent consumer perception (14, 23). This study raises critical concerns about the conceptual foundations of brand personality research. The authors of this study primarily question whether consumers genuinely process brands as humanlike entities or if such characterizations arise primarily from methodological influences. Understanding this distinction is essential for refining the theoretical underpinnings of brand personality and ensuring that brand-related personality constructs align with actual consumer cognition rather than artificially induced anthropomorphic associations (14, 23, 27).

### Criticism regarding applicability of Aaker's scale across product categories

One of the major criticisms of Aaker's scale is associated with non-generalizability of Aaker's scale across different product categories (17). Austin and his colleagues examined the measurement properties of Aaker's brand personality structure for individual brands (i.e., nine restaurant brands) within a product category (i.e., upscale dining restaurant, casual dining, and quick service), as well as brands aggregated within the same category. Their results revealed that the confirmatory factor analysis (CFA) model did not provide a satisfactory fit for disaggregate data or aggregated data sets (17).

In the sport context, Ross (3) examined the generalizability of Aaker's factor structure. He hypothesized that the scale would be applicable in the field of sport. Ross (3) assessed the brand personality of an intercollegiate basketball team using Aaker's 42 items. Although the reliability results ranged from.7 to.91, each of these dimensions met the suggested threshold by Nunnally and Bernstein (28), the CFA model of his study fails to produce a satisfactory fit for the sport brand (3). Concerning the non-generalizability and non-replicability of Aaker's scale for brands across different categories, several methodological reasons have been identified to explain these problems (13, 27, 29, 30).

One of the potential methodological limitations of Aaker's scale is the use of metaphors as descriptors of brand personality in research (29). The term “metaphor” refers to “a process of transposing a word meaning to another meaning using either an analogy or an implicit comparison” (31). Through direct and symbolic analogies, a brand as a valued material possession can be considered as a person such as a friend, lover, or even part of ourselves (i.e., extended self) (32).

Consumers tend to express themselves through possessions and utilize material possessions to seek, communicate, confirm, and reinforce their identities (32, 33). For example, if one owns an invaluable brand (e.g., luxury car brand) that serves a symbolic or self-expressive function presenting oneself, in this case, the brand can be considered as an extension of oneself (32). In such circumstances, if brands are actually perceived as human-like entities, applying the human personality measure to brands may generate different results from Caprara et al.'s (15) study (27).

Therefore, it is essential to investigate whether individuals perceive a brand as humanlike when assessing its brand personality. If consumers do not perceive a brand as a humanlike entity, the personality traits used in Caprara et al.'s (15) study may be interpreted differently by respondents, deviating from the original meaning of the items (27). For instance, when we use the term “wholesome” to describe a food brand (e.g., an Italian pasta brand) and a friend, the meanings may not be identical (26, 27).

Low and Lamb (34) argued that “the generalizability of the brand personality is limited because many brands are not personality brands, and no protocol is given to adapt the scale” (p. 352). Consequently, when applying human personality traits to describe brand personality, it is crucial to consider whether consumers perceive brands as human entities (e.g., athlete brands, sport teams as human entities composed of a group of athlete brands, politician brands) or at least as humanlike entities (e.g., Air Jordan).

A human brand can be defined as “any well-known persona who is the subject of marketing communications efforts” (35). Arai et al. (36) extended this concept to the realm of sport while suggesting that all individual athletes can be considered as brands having “a name, distinctive appearance, and a personality” (p. 98). Human brands, including athletes and sport teams, can embody a unique combination of personal characteristics, achievements, and public personas (35, 37). These differences are readily observable and interpretable by consumers who use language to capture and convey the essence of these sport brands. The lexical approach assumes that all significant individual differences and personality traits are encoded in the natural language people use. This concept explains why brand personality studies can adopt a lexical approach, as it is founded on systematically categorizing human characteristics through the language used by sport consumers as a theoretical and methodological framework (30).

Researchers studying brand personality should assess consumers' actual perceptions of brand personality based on their experiences or memories of specific brands instead of exploring projected human characteristics of brands not derived from their genuine recollections (14). In addition, sport enthusiasts typically exhibit highly committed and emotionally invested relationships with their favored sport brands. This strong connection not only fosters a profound sense of loyalty and identification, but also ensures that assessments of brand personality are both meaningful and reflective of the deep-seated bonds that fans share with their favorite sport brands, enabling precise and insightful evaluations of brand personality dimensions. Historically, investigations into sport brand personality have predominantly relied on convenience samples, including undergraduate students, mid-Atlantic college basketball fans, German online panel participants, sport fans in Greece, Netball Victoria members (players), Australian Generation Y consumers, and spectators in New Zealand (1–4, 30, 38–41). Despite this diversity in various sport, there remains a critical need to recruit a broad and diverse participant pool encompassing different demographics, geographies, and backgrounds, with a particular focus on the U.S. market (5).

### Criticism regarding the descriptor selection problem

A third criticism of the descriptor selection problem is the lack of a theoretical basis to select a set of representative personality traits when finding any major brand personality dimensions (13, 27, 30). Before addressing this criticism in detail, it is essential to provide some background on the Big Five and HEXACO models, as they are central to our discussion.

The Big Five and HEXACO models represent foundational frameworks in personality psychology, offering a comprehensive structure for categorizing human personality traits. The Big Five model, also known as the Five Factor Model, posits five broad dimensions of personality: Extraversion, Agreeableness, Conscientiousness, Emotional Stability, and Intellect/Imagination. This model emerged from decades of research in psychology, initially through factor analyses of personality adjectives and statements in the English language, which later expanded to multiple languages and cultures, highlighting its universal applicability (42. 74).

Similarly, the HEXACO model extends the Big Five framework by adding a sixth dimension, Honesty-Humility, and reinterpreting some of the traditional five dimensions. It was developed through lexical studies that included languages not previously analyzed in Big Five research, providing a broader, cross-cultural perspective on personality structure (43).

In personality psychology, the lexical approach is a theoretical and methodological foundation for identifying representative personality traits across various languages and cultures. This approach posits that all significant individual differences are encoded within the natural language, leading to the derivation of both the Big Five personality factors and the HEXACO model based on personality lexicons in various languages and cultures (44–46).

Drawing from the lexical approach, researchers in the field of sport brand personality aim to establish a set of representative traits that accurately capture the dimensions of major sport brands' personalities. However, the criticism arises from the challenge of selecting these traits without a universally accepted theoretical basis, similar to the robust foundations provided by the lexical approach to human personality. This lack of theoretical grounding complicates the descriptor selection process, highlighting the need for a more structured approach to identify sport brand personality dimensions that are as theoretically sound and universally applicable as those found in personality psychology.

However, contrary to the cross-cultural generalizability of the Big Five structure or HEXACO model in different languages, previous sport brand personality studies have generated different brand (team) personality dimensions that have shown no resemblance either with the Aaker's brand personality structure or with the Big Five structure or HEXACO model (see Table 1) (13, 47).

**Table 1:** A comparison of (sport) brand (team) personality and human personality (Big five/HEXACO) structures.

Author (s)	Conceptual Definition of BP	Adjective-selection procedure by	Adjectives	Dimensions of Personality	Dimensions related to Human Personality Factors
Human Personality Big Five (42)		Lexical approach	Personality traits	1. Agreeableness 2. Conscientiousness 3. Extraversion 4. Emotional Stability (Neuroticism) 5. Openness/Intellect/Imagination	
Human Personality HEXACO (48)		Lexical approach	Personality traits	1. Agreeableness 2. Extraversion 3. Conscientiousness 4. Emotionality 5. Openness to Experience 6. Honesty-Humility	
Aaker (12) Consumer brands	Aaker's (12) definition		Personality and non-personality traits	1. Sincerity 2. Excitement 3. Competence 4. Sophistication 5. Ruggedness	
Geuens et al. (13) Consumer brands	Azoulay & Kapferer's (15)	Lexical approach	Personality traits	1. Conscientiousness/Responsibility 2. Extraversion/Activity 3. Emotional Stability/Emotionality 4. Agreeableness/Aggressiveness 5. Openness/Simplicity	C (Big 5 or HEXACO) X (Big 5 or HEXACO) E (Big 5 or HEXACO) A (Big 5 or HEXACO) O (Big 5 or HEXACO)
Ross (4) Sport brands (Intercollegiate team sports)	Aaker's (12) definition		Personality and non-personality traits	1. Sincerity 2. Sophistication 3. Excitement 4. Ruggedness 5. Competence	
Braunstein & Ross (2) Sport brands (Professional team sports)	Aaker's (12) definition		Personality and non-personality traits	1. Success 2. Sophistication 3. Sincerity 4. Rugged 5. Community-driven 6. Classic	
Heere (3) Sport brands (Professional team sports)			Personality and non-personality traits	1. Game related 2. Event related	
Lee and Cho (39) Sport brands (Sporting events (e.g., Super Bowl, NBA Playoffs, Olympic Games)	Aaker's (12) definition		Personality and non-personality traits	1. Diligence 2. Uninhibitedness 3. Fit 4. Tradition 5. Amusement	
Tsiotsou (7) Sport brands (Professional team sports)	Azoulay & Kapferer's (15) definition		Personality and non-personality traits	1. Competitiveness 2. Prestige 3. Morality 4. Authenticity 5. Credibility	
Schade et al. (5) (Professional Sport Clubs)	Azoulay & Kapferer's (15)		Personality traits	1. Extraversion 2. Rebellious 3. Open-Mindedness 4. Conscientiousness	
Kang et al. (30) (Professional sport league)	Azoulay & Kapferer's (15)	Lexical approach	Personality traits	1. Agreeableness 2. Extraversion/Emotionality 3. Openness 4. Conscientiousness 5. Honesty	A (Big 5 or HEXACO) X/E (Big 5 or HEXACO) O (Big 5 or HEXACO) C (Big 5 or HEXACO) H (HEXACO)
Stadler Blank et al. (6) (Professional sport teams in the United States and United Kingdom.	Aaker's (12) definition		Personality and non-personality traits	1. Success 2. Talent 3. Entertainment 4. Dedication 5. Admiration 6. Care	

A, Agreeableness; C, Conscientiousness; E, Emotionality; H, Honesty-Humility; O, Openness to Experience (Intellect-Imagination); X, Extraversion.

As shown in Table 1, brand personality studies developed by Aaker's definition and methodology exhibit numerous variations on the facets or factors in their models compared to the studies conducted based on the underlying conceptualization and methodology by Azoulay and Kapferer's (16) study or human personality studies based on the lexical approach (27, 29).

Previous research has identified several potential factors influencing outcomes in scale development, such as the exclusion of negatively keyed items (49), the examination of consumers' perception of unfamiliar brands, the use of aggregated data that excludes within-brand variance, the influence of culture-based brand perceptions, and the reliance on nonipsatized scores rather than within-subject standardized ratings (i.e., ipsatized scores) (13, 17, 27, 29, 49, 50).

While these circumstances might explain some of the variations in brand personality, the most likely explanation for the discrepancies across brand personality studies can be attributed to the methods used for selecting adjectives (27). According to the lexical approach in personality psychology, which focuses on human personality traits, adjective selection in human personality studies should involve terms that describe normal personality variation.

Thus, lexical studies should not include highly evaluative terms, temporary states, or physical characteristics that do not represent relatively enduring patterns of typical behavioral tendencies to identify personality structures (44). On the other hand, Aaker's (12) study, followed by countless brand personality studies, includes numerous temporary states or highly evaluative terms in item generation (e.g., good-looking, glamorous, popular, satisfying, successful) in their subjective criteria. This inclusion raises concerns about whether such terms capture stable brand personality traits or merely reflect consumer perceptions influenced by temporary marketing efforts. The mere administration without excluding such adjectives in item selection might influence the results of studies to identify brand personality structures (18). Consequently, the inappropriate selection of descriptors may distort the obtained factor structure of brand personality (44).

### Criticism regarding to the potential problem of brand selection

Brand personality research has traditionally selected well-known brands for scale development, assuming their higher media exposure, perceived quality, and strong brand image foster meaningful customer-brand relationships (12, 13, 47). According to consumer-brand relationship theory, consumers can develop long-term, committed, and intense relationships with various products and services, including food, clothing, tools, and household technologies (8, 51). However, not all well-known brands necessarily serve as strong, committed consumer partners.

Judging brand personality accurately may depend on the strength of the consumer-brand relationship, as research suggests that accuracy in assessing others' personalities improves with closer relationships (52, 53). Consumers who maintain strong connections with a brand may be better positioned to evaluate its brand personality more precisely. Therefore, selecting consumers' favorite brands, those with high levels of commitment and strong consumer-brand relationships, may help address this potential issue (29).

Unlike traditional humanized brands, where human personality is strategically crafted through marketing efforts and reinforced by ongoing humanization strategies, sport teams, as sport brands, can develop personality traits through a combination of team history, identities of its players, fan culture, organizational values and cultures, on-field performance, and long-standing traditions (1, 4–6, 54). Therefore, we posit that this study is well-positioned to address the criticisms of brand selection in traditional brand personality research by focusing on sport teams as sport brands. The sport teams may inherently encompass all types of human brand associations, including brand representatives (e.g., athletes, coaches), brand characters (e.g., team mascot, iconic players, team identities), and brand users (e.g., sport fans, fan communities) (14).

In psychology, team personality composition refers to the collective personality traits of a team, typically derived from the aggregation, similarity, or configuration of individual team members' traits (55, 56). Studying team personality composition provides valuable insights into how collective personality traits influence team performance and organizational effectiveness (55, 56). This study adopts Barrick et al.'s (55) perspective, which emphasizes the mean-level composition model, where a team's personality is understood as the aggregate of its members' personality traits. While sport teams are composed of multiple individuals (players, coaches, managers), their collective personality can be perceived as a unified entity by fans and stakeholders, as Barrick et al.'s model suggests that individual personality traits aggregate to reflect overall team characteristics. However, most studies on team personality composition have focused on self-assessments by team members, limiting their applicability to research examining external perceptions of team characteristics. Therefore, personality traits in personality psychology may not fully capture sport team characteristics, as they were not specifically designed for this context. To address this limitation, we consulted sport management faculty to identify the most relevant and applicable traits for describing sport teams.

Using a lexical approach, we incorporated these expert-selected traits to define perceived team personality composition (PTPC) as externally attributed personality traits of a sport team, as perceived by fans, stakeholders, and the public. This approach ensures that the trait list accurately reflects the distinct attributes of sport teams while enhancing its relevance and applicability. Moreover, this study expands existing research by bridging the gap between traditional brand personality and sport team personality studies. Through a lexical approach, it further examines how a sports team's perceived personality emerges from its structural elements and the collective traits of its fans.

Given this conceptual, theoretical, and methodological foundation of brand personality, the present study developed research questions to focus on the following:

R1: What is the main structure of perceived team personality traits in sport when using a lexical approach as a conceptual, theoretical, and methodological basis?
R2: Are dimensions of perceived team personality traits in sport similar to the Big Five or HEXACO structure based on the lexical approach?

## Method

### Selection of a representative set of human personality traits applicable and relevant to sport teams

To compile a comprehensive list of human personality traits applicable to sport team personality composition, we first reviewed established research on brand (team) personality and personality psychology. Since previous brand (team) personality studies in sport have included personality and non-personality traits, we adopted a stricter criterion to focus exclusively on descriptors that align with human personality traits. To achieve this, we applied a lexical approach using Allport and Odbert's (57) compilation of 4,504 human personality traits and Norman's (58) list of 2,800 descriptive human personality terms as inclusion and exclusion criteria. Additionally, we focused solely on adjectives, as prior lexical research has demonstrated that adjectives are the primary and most effective means of describing personality attributes (44, 59). Unlike nouns and verbs, adjectives inherently denote properties that can be expressed in varying degrees, making them particularly suitable for characterizing personality traits (44).

We removed any trait not found in at least one of these lexical studies. For example, in Schade et al.'s (4) study, fighting spirit and alternative were excluded because they did not appear in either Allport and Odbert's (57) or Norman's (58) lists, indicating that they do not align with established human personality descriptors in personality research. This systematic approach ensured that our selection remained consistent with the lexical foundation of personality research and eliminated potential biases by non-personality traits often included in prior sport brand (team) personality studies. Given the extensive overlap among studies, we quantified the frequency of adjectives appearing in previous research. Some traits, such as sociable, shy, honest, gentle, cheerful, and anxious, appeared more than 26 times. To refine the list for expert evaluation, we retained only adjectives that appeared in multiple lexical studies, resulting in a final set of 499 adjectives.

To further refine the list, a panel of seven sport management faculty (6 men, 1 woman; *M*_age_ = 40.9, SD_age_ = 7.4), all native English speakers with at least five years of experience as professors in higher education, assessed the relevance and applicability of each trait to sport teams using a four-point scale (1 = not relevant/applicable, 4 = highly relevant/applicable). The intraclass correlation coefficient (ICC) for single measures was.418, indicating moderate agreement among individual raters. The ICC for average measures was.834, which suggested strong reliability when ratings were aggregated. Based on the scores of the panels, traits with a mean score of 3.0 or higher were retained. While negative adjectives generally had lower scores, we included 17 traits when their corresponding positive counterparts met the threshold. (e.g., disorganized, insincere, undisciplined, uncommunicative). This decision aligned with previous research suggesting that excluding negatively keyed items can introduce methodological limitations in scale development, such as response bias and reduced conceptual coverage of the construct (49). Including positive and negative traits might ensure a more balanced representation of team personality attributes, accounts for the full spectrum of consumer perception, and mitigates potential issues using only positively framed descriptors.

### Participants and procedures

This study recruited an online sample of adult participants (*N* = 522) for data collection through Amazon's Mechanical Turk. Using a 7-point Likert-type scale (1 = strongly disagree, 7 = strongly agree), a total of 516 respondents rated how relevant and applicable the 99 human personality traits were to their favorite major professional sport teams (e.g., Dallas Cowboys, New York Yankees, Chicago Bulls, LA Galaxy). Prior to describing the sport team personality traits for their favorite teams, we informed research participants that the selected sport team should be familiar, relevant, and meaningful to them. The sample included 254 female participants (49.2%) and 262 (50.8%) male participants. The respondents' mean average age was 37.4 (SD = 11.2) and ranged from 19 to 75. Fifty-four and one-half percent had obtained a four-year college degree or advanced degree. The majority of participants identified as National Football League (NFL) fans (*N* = 257), followed by Major League Baseball (MLB) fans (*N* = 122), National Basketball Association (NBA) fans (*N* = 81), National Hockey League (NHL) fans (*N* = 36), and fans of other sports [e.g., Major League Soccer (MLS), *N* = 6]. This study recruited a nationwide sample of sport fans across the United States, excluding Alaska, Hawaii, North Dakota, Rhode Island, and Wyoming, thereby covering 45 states. The data indicated a significant relationship (*r* = .902) between the number of subjects in each state and the state's population. In addition, the data encompassed the majority of professional sport teams. However, the data did not include three teams (Tennessee Titans, Washington Commanders, Los Angeles Rams) from the National Football League (NFL), six teams from Major League Baseball (MLB), eight teams from the National Basketball Association (NBA), and 18 teams from the National Hockey League (NHL). Notably, most teams (e.g., Toronto Blue Jays, Toronto Raptors) based in Canada were not found, which may be due to the data collection's focus on United States territory.

### Data analysis

Before conducting a principal component analysis (PCA) to identify the underlying dimensions of team personality, we examined intraclass correlation coefficients (ICC) to assess the consistency of individual and aggregated ratings among raters of personality traits for each team among raters. Given that the number of raters varied across teams, a one-way random effects model was used for ICC calculations (see Table 2). Table 2 shows the ICC values ranged from poor to excellent reliability across the 57 teams.

**Table 2:** Intraclass correlation coefficients (ICC) and descriptive statistics for professional sport teams.

League	Team	*N*	*M*	*SD*	ICC (1, 1)	ICC (1, *k*)	Interpretation	*W*	*p*	Level of Agreement
MLB	Atlanta Braves	10	5.492	.142	.656	.950	Excellent	.225	<.001	Fair agreement
	Boston Red Sox	13	5.235	.178	.605	.952	Excellent	.156	<.001	Slight agreement
	Chicago Cubs	11	4.845	.170	.508	.919	Excellent	.136	<.001	Slight agreement
	Cincinnati Reds	5	4.598	.184	.362	.739	Good	.187	<.001	Slight agreement
	Detroit Tigers	7	5.136	.153	.584	.908	Excellent	.163	<.001	Slight agreement
	Houston Astros	3	4.997	.820	.144	.335	Poor	.376	<.001	Fair agreement
	Kansas City Royals	4	5.134	.114	.642	.878	Good	.110	<.001	Slight agreement
	Los Angeles Dodgers	5	4.921	.339	.419	.783	Good	.250	<.001	Fair agreement
	New York Mets	6	4.926	.368	.361	.819	Good	.233	<.001	Fair agreement
	New York Yankees	21	4.897	.183	.453	.946	Excellent	.129	<.001	Slight agreement
	Philadelphia Phillies	6	4.790	.315	.340	.756	Good	.189	<.001	Slight agreement
	San Francisco Giants	5	4.956	.091	.660	.906	Excellent	.107	<.001	Slight agreement
	St. Louis Cardinals	5	5.097	.127	.611	.887	Good	.131	<.001	Slight agreement
	Texas Rangers	3	5.532	.205	.797	.922	Excellent	.282	<.001	Fair agreement
NBA	Boston Celtics	6	5.315	.312	.577	.891	Good	.278	<.001	Fair agreement
	Chicago Bulls	17	5.256	.380	.374	.910	Excellent	.226	<.001	Fair agreement
	Cleveland Cavaliers	8	5.429	.052	.674	.943	Excellent	.082	<.001	Slight or no agreement
	Golden State Warriors	4	5.240	.364	.504	.803	Good	.305	<.001	Fair agreement
	Los Angeles Lakers	10	5.254	.177	.592	.936	Excellent	.143	<.001	Slight agreement
	New York Knicks	4	5.361	.290	.451	.766	Good	.263	<.001	Fair agreement
	Orlando Magic	3	5.162	.553	.560	.792	Good	.390	<.001	Fair agreement
	San Antonio Spurs	6	5.064	.101	.601	.900	Excellent	.047	<.001	Slight or no agreement
	Utah Jazz	3	5.226	.004	.661	.854	Good	.012	<.001	Slight or no agreement
NFL	Arizona Cardinals	3	5.377	.153	.735	.893	Good	.242	<.001	Fair agreement
	Atlanta Falcons	3	5.077	.324	.387	.654	Moderate	.234	<.001	Fair agreement
	Baltimore Ravens	6	5.665	.065	.756	.949	Excellent	.172	<.001	Slight agreement
	Buffalo Bills	3	4.764	.433	.151	.348	Poor	.086	<.001	Slight or no agreement
	Carolina Panthers	14	5.203	.569	.461	.923	Excellent	.292	<.001	Fair agreement
	Chicago Bears	13	5.066	.287	.417	.903	Excellent	.214	<.001	Fair agreement
	Cincinnati Bengals	4	5.003	.419	.242	.561	Moderate	.230	<.001	Fair agreement
	Cleveland Browns	6	4.791	.494	.375	.783	Good	.229	<.001	Fair agreement
	Dallas Cowboys	27	5.077	.281	.439	.955	Excellent	.204	<.001	Fair agreement
	Denver Broncos	19	5.123	.196	.530	.955	Excellent	.191	<.001	Slight agreement
	Detroit Lions	3	4.727	.187	.157	.358	Poor	.113	<.001	Slight agreement
	Green Bay Packers	12	5.006	.164	.557	.938	Excellent	.115	<.001	Slight agreement
	Houston Texans	4	5.361	.705	.498	.799	Good	.296	<.001	Fair agreement
	Indianapolis Colts	3	5.441	.055	.721	.886	Good	.134	<.001	Slight agreement
	Jacksonville Jaguars	3	5.178	.221	.558	.791	Good	.184	<.001	Slight agreement
	Kansas City Chiefs	3	5.407	.055	.710	.880	Good	.104	<.001	Slight agreement
	Las Vegas Raiders	7	5.185	.270	.416	.833	Good	.252	<.001	Fair agreement
	Miami Dolphins	4	4.669	.334	.210	.516	Moderate	.246	<.001	Fair agreement
	Minnesota Vikings	10	4.907	.336	.321	.826	Good	.211	<.001	Fair agreement
	New England Patriots	13	5.324	.074	.604	.952	Excellent	.133	<.001	Slight agreement
	New Orleans Saints	14	5.299	.436	.349	.883	Good	.263	<.001	Fair agreement
	New York Giants	7	5.229	.210	.588	.909	Excellent	.237	<.001	Fair agreement
	New York Jets	4	4.960	.543	.256	.579	Moderate	.272	<.001	Fair agreement
	Philadelphia Eagles	12	4.911	.235	.481	.918	Excellent	.159	<.001	Slight agreement
	Pittsburgh Steelers	16	5.270	.173	.624	.964	Excellent	.191	<.001	Slight agreement
	San Diego Chargers	3	4.949	.184	.352	.620	Moderate	.134	<.001	Slight agreement
	San Francisco 49ers	13	4.923	.243	.463	.918	Excellent	.210	<.001	Fair agreement
	Seattle Seahawks	19	5.200	.081	.636	.971	Excellent	.104	<.001	Slight agreement
	Tampa Bay Buccaneers	5	5.129	.183	.378	.752	Good	.172	<.001	Slight agreement
NHL	Boston Bruins	3	5.552	.120	.681	.865	Excellent	.173	<.001	Slight agreement
	Chicago Blackhawks	3	5.273	.020	.699	.875	Good	.014	.253	No agreement
	Detroit Red Wings	4	4.851	.165	.374	.705	Moderate	.131	<.001	Slight agreement
	Pittsburgh Penguins	7	5.100	.204	.531	.888	Good	.200	<.001	Fair agreement
	Tampa Bay Lightening	3	5.300	.230	.640	.842	Good	.299	<.001	Fair agreement

Note. Fifty-seven teams are listed in alphabetical order. *N* rater refers to the number of raters for each team. ICC (1,1) represents interrater reliability for individual raters, while ICC (1, *k*) represents reliability when averaging across raters. Mean (*M*) values indicate the central tendency of responses, while variance (*SD*) reflects the dispersion of ratings. Interpretation follows Koo & Li (60): ICC < 0.50 = poor reliability, 0.50–0.75 = moderate reliability, 0.75–0.90 = good reliability, >0.90 = excellent reliability. Only teams (*N* = 57) with at least three raters were included. Kendall's W (*W*) indicates the level of agreement among raters. Interpretation follows Landis & Koch (61): *W* < 0.20 = slight agreement, 0.20–0.40 = fair agreement, 0.40–0.60 = moderate agreement, 0.60–0.80 = substantial agreement, >0.80 = almost perfect agreement.

Of the 57 teams, 24 teams (42.1%) demonstrated excellent reliability [ICC (1, *k*) > 0.90], 24 teams (42.1%) exhibited good reliability [ICC (1, *k*) = 0.75–0.90], 6 teams (10.5%) showed moderate reliability [ICC (1, *k*) = 0.50–0.75], and only 3 teams (5.3%) had poor reliability [ICC (1, *k*) < 0.50]. The teams with the highest reliability scores included the Dallas Cowboys [ICC (1, *k*) = 0.955], New England Patriots [ICC (1, *k*) = 0.952], and Atlanta Braves [ICC (1, *k*) = 0.950], which indicated that fan ratings were highly consistent for these teams' perceived personalities. In contrast, teams such as the Buffalo Bills [ICC (1, *k*) = 0.361] and Houston Astros [ICC (1, *k*) = 0.144] exhibited poor reliability, suggesting more significant variability in how fans perceived their personality traits. In addition, Table 3 shows descriptive statistics (mean ± SD) for Six Team Personality Dimensions by Professional Sport Teams.

**Table 3:** Descriptive statistics (mean ± SD) for six team personality dimensions by professional sport teams.

League	Team	*N*	FH	BT	EX	CA	GC	AO
MLB	Atlanta Braves	10	6.32 ± .73	6.52 ± .54	6.29 ± .55	4.73 ± .46	6.10 ± .68	5.88 ± .68
	Boston Red Sox	13	6.00 ± .46	6.19 ± .55	5.92 ± .47	4.81 ± .38	5.93 ± .47	5.56 ± .92
	Chicago Cubs	11	5.57 ± .65	5.56 ± .47	5.70 ± .56	4.88 ± .58	5.46 ± .53	4.90 ± .72
	Cincinnati Reds	5	5.10 ± .47	5.10 ± .47	5.10 ± .47	5.10 ± .47	5.10 ± .47	5.10 ± .47
	Detroit Tigers	7	6.00 ± .65	5.73 ± .95	5.68 ± .55	4.33 ± .49	5.87 ± .47	5.40 ± .65
	Houston Astros	3	5.33 ± 1.50	5.80 ± 1.44	5.24 ± 1.08	4.67 ± .67	5.51 ± 1.38	5.29 ± 1.45
	Kansas City Royals	4	6.23 ± .34	6.28 ± .67	6.07 ± .16	4.46 ± .42	5.99 ± .46	5.31 ± 1.02
	Los Angeles Dodgers	5	5.26 ± 1.14	5.88 ± .86	5.60 ± .86	4.57 ± .30	5.59 ± .98	4.69 ± .86
	New York Mets	6	5.34 ± 1.04	5.45 ± 1.17	5.67 ± .80	4.60 ± .51	5.43 ± .90	5.15 ± .88
	New York Yankees	21	5.35 ± .86	5.69 ± .92	5.43 ± .68	4.51 ± .54	5.59 ± .59	5.10 ± .69
	Philadelphia Phillies	6	5.55 ± .79	5.13 ± 1.38	5.49 ± .83	4.78 ± .60	5.11 ± .79	5.18 ± .70
	San Francisco Giants	5	6.18 ± .41	6.02 ± .18	5.32 ± .21	4.60 ± .25	5.92 ± .42	5.18 ± .67
	St. Louis Cardinals	5	6.02 ± .51	5.80 ± .53	5.51 ± .47	4.77 ± .72	5.89 ± .41	5.46 ± .75
	Texas Rangers	3	6.67 ± .31	6.27 ± .51	6.21 ± .38	4.89 ± .48	6.44 ± .39	6.27 ± .75
NBA	Boston Celtics	6	5.77 ± .62	6.45 ± .55	5.76 ± .81	4.25 ± .33	5.77 ± .87	5.83 ± .73
	Chicago Bulls	17	5.67 ± 1.04	5.85 ± 1.04	5.74 ± .96	4.56 ± .82	5.69 ± .88	5.55 ± .99
	Cleveland Cavaliers	8	5.85 ± .62	6.53 ± .33	6.03 ± .28	4.83 ± .35	6.12 ± .27	6.01 ± .49
	Golden State Warriors	4	5.55 ± 1.05	5.88 ± 1.01	5.64 ± .75	4.58 ± .35	5.82 ± .95	5.81 ± 1.16
	Los Angeles Lakers	10	5.83 ± .65	6.39 ± .38	5.92 ± .60	4.55 ± .45	5.98 ± .95	5.62 ± .63
	New York Knicks	4	5.53 ± 1.00	6.30 ± .50	5.91 ± .39	4.13 ± .25	5.64 ± .78	5.74 ± 1.00
	Orlando Magic	3	5.87 ± 1.10	5.73 ± .76	5.67 ± .71	5.44 ± .69	5.78 ± .84	5.94 ± .92
	San Antonio Spurs	6	6.48 ± .48	5.73 ± .65	4.58 ± 1.56	4.78 ± .87	6.22 ± .28	5.69 ± .67
	Utah Jazz	3	5.90 ± .46	6.47 ± .15	5.94 ± .34	4.72 ± .42	6.04 ± .34	4.96 ± .24
NFL	Arizona Cardinals	3	5.33 ± .70	6.33 ± .55	5.79 ± .83	4.39 ± .19	5.92 ± .36	5.47 ± .54
	Atlanta Falcons	3	5.73 ± 1.22	5.83 ± .59	6.12 ± .53	4.67 ± .58	5.68 ± 1.19	4.77 ± 1.75
	Baltimore Ravens	6	6.52 ± .49	6.88 ± .19	6.35 ± .31	4.19 ± .27	6.50 ± .24	6.13 ± .60
	Buffalo Bills	3	5.77 ± .31	4.77 ± 1.74	5.58 ± 1.05	4.89 ± .42	4.71 ± 1.52	4.80 ± 1.07
	Carolina Panthers	14	5.67 ± 1.38	6.00 ± 1.27	5.83 ± 1.02	4.45 ± 1.11	5.70 ± 1.20	5.45 ± 1.10
	Chicago Bears	13	5.36 ± .97	5.74 ± 1.05	5.66 ± .86	4.73 ± .72	5.53 ± .81	5.14 ± .99
	Cincinnati Bengals	4	5.33 ± 1.40	5.80 ± 1.22	5.50 ± .71	4.21 ± .21	5.12 ± 1.48	5.22 ± 1.06
	Cleveland Browns	6	5.43 ± .67	5.52 ± 1.26	5.42 ± .69	4.39 ± .54	5.31 ± .96	4.94 ± .86
	Dallas Cowboys	27	5.39 ± .85	5.93 ± .89	5.70 ± .81	4.75 ± .70	5.60 ± .83	5.12 ± 1.00
	Denver Broncos	19	5.60 ± .82	6.02 ± .57	5.68 ± .55	4.53 ± .58	5.68 ± .71	5.08 ± .78
	Detroit Lions	3	5.50 ± .66	5.17 ± .45	4.76 ± .56	4.78 ± .35	4.64 ± .21	4.82 ± .41
	Green Bay Packers	12	5.66 ± .47	5.92 ± .73	5.64 ± .51	4.63 ± .43	5.66 ± .51	5.08 ± .91
	Houston Texans	4	5.90 ± 1.22	6.20 ± 1.53	5.77 ± 1.18	4.33 ± .47	5.87 ± 1.32	5.56 ± 1.15
	Indianapolis Colts	3	5.87 ± .57	6.33 ± .49	5.94 ± .55	4.28 ± .19	6.17 ± .33	5.67 ± .66
	Jacksonville Jaguars	3	6.20 ± .87	5.80 ± 1.31	6.15 ± .45	4.56 ± .35	6.08 ± .58	5.53 ± .89
	Kansas City Chiefs	3	6.40 ± .70	6.83 ± .06	6.09 ± .40	4.56 ± .26	6.39 ± .30	5.75 ± .18
	Las Vegas Raiders	7	5.00 ± .86	6.39 ± .64	6.05 ± .66	4.26 ± .60	5.38 ± .66	5.53 ± .74
	Miami Dolphins	4	5.35 ± .86	5.23 ± 1.15	4.84 ± .78	4.42 ± .55	5.08 ± 1.10	4.50 ± .57
	Minnesota Vikings	10	5.26 ± .88	5.60 ± 1.04	5.22 ± .81	4.55 ± .37	5.30 ± .79	4.72 ± .95
	New England Patriots	13	5.19 ± .62	6.17 ± .57	5.88 ± .46	4.60 ± .47	5.98 ± .45	5.58 ± .54
	New Orleans Saints	14	5.54 ± 1.24	6.05 ± 1.23	5.96 ± .59	4.52 ± .80	5.64 ± .77	5.43 ± 1.04
	New York Giants	7	5.99 ± .82	6.11 ± .72	5.64 ± .77	4.07 ± .58	5.95 ± .66	5.71 ± .60
	New York Jets	4	4.93 ± 1.19	5.78 ± 1.45	5.66 ± 1.07	4.17 ± .27	5.58 ± 1.08	5.07 ± .99
	Philadelphia Eagles	12	5.33 ± .73	5.96 ± .66	5.70 ± .67	4.44 ± .46	5.49 ± .68	4.78 ± .87
	Pittsburgh Steelers	16	5.79 ± .86	6.28 ± .68	5.86 ± .54	4.62 ± .50	6.02 ± .55	5.47 ± .79
	San Diego Chargers	3	5.17 ± 1.00	5.90 ± .56	5.85 ± .29	4.56 ± .19	5.14 ± .96	4.94 ± .33
	San Francisco 49ers	13	5.31 ± .77	5.86 ± .70	5.55 ± .72	4.24 ± .56	5.42 ± .94	5.05 ± .88
	Seattle Seahawks	19	5.67 ± .53	6.05 ± .44	5.91 ± .56	4.49 ± .33	5.85 ± .43	5.44 ± .51
	Tampa Bay Buccaneers	5	5.60 ± .65	6.24 ± .74	5.86 ± .74	4.77 ± .67	5.23 ± .80	5.40 ± 1.10
NHL	Boston Bruins	3	6.43 ± .51	6.53 ± .57	6.03 ± .76	4.28 ± .10	6.22 ± .45	5.77 ± .97
	Chicago Blackhawks	3	5.97 ± .47	6.23 ± .12	6.03 ± .34	4.28 ± .19	6.14 ± .31	4.98 ± .64
	Detroit Red Wings	4	5.13 ± .77	5.75 ± .61	5.32 ± .47	4.67 ± .41	5.21 ± .89	5.07 ± .66
	Pittsburgh Penguins	7	5.86 ± .79	5.93 ± .69	5.77 ± .59	4.71 ± .30	5.85 ± .76	5.22 ± .94
	Tampa Bay Lightening	3	5.70 ± .82	6.50 ± .44	5.82 ± .60	4.44 ± .10	5.81 ± .73	5.29 ± .46

Note. Fifty-seven teams are listed in alphabetical. AO, Adaptive Openness; BT, Braveness-Toughness; EX, Energetic Extraversion; FH, Fair-Minded Honesty; GC, Coal-Oriented Conscientiousness; CA, Combative Aggression.

While the dataset includes teams from multiple leagues, the primary objective was to identify the underlying factors of team personality using individual-level ratings consistent with established methods in personality psychology. Given this focus, assessing within-team agreement was unnecessary, as the analysis did not involve aggregating data at the team level. Instead, the study examined the structure of perceived team personality based on individual responses across teams and leagues. To further investigate the structure of team personality traits, the researchers analyzed the set of 99 team personality traits by performing a series of principal component analyses from the first one-factor solution to the six-factor solution (18, 62). This hierarchical analysis assessed whether sport team personality dimensions align with the Big Five or HEXACO factors. Accordingly, multiple analyses were conducted to examine the hierarchical emergence of components through one to six-factor solutions.

Additionally, we conducted the factor analyses by using within-subject standardized ratings (ipsatized responses) on the 99 adjectives (18). As a common method in personality psychology, the purpose of the ipsatization (Z standardization) was “to prevent the potential distortion of factor analytic results that may result from individual differences in the overall elevation or extremity of responses to items” (63). This statistical procedure controls for individual differences in the elevation and extremity of participants' scores (18) and is calculated as follows:

$$z_{i\dot{\;j}}=\frac{x_{ij}-{\bar{x}}_{i}}{s_{i}}$$

where $\;z_{i\dot{\;j}}$ is ipsatized score for individual *i* on item $j;\;x_{ij}$ represents raw score for individual *i* on item j; ${\bar{x}}_{i}$ represents mean of all items for individual *i*; $s_{i}$ represents standard deviation of all items for individual *i*.

## Results

**Figure 1:**
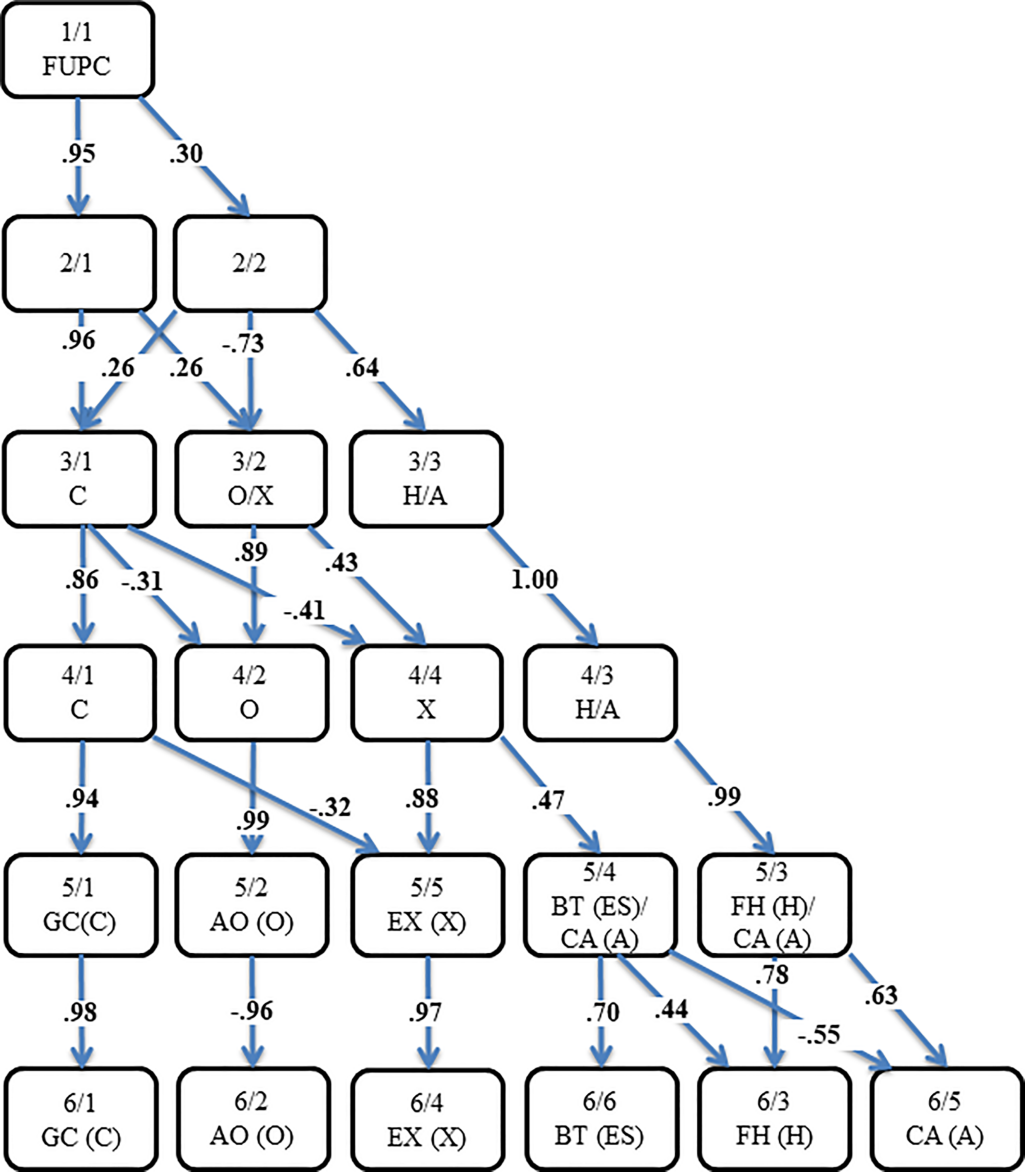
Component solutions obtained from 516 ratings on 99 brand personality traits. The examination focuses on correlations between components of adjacent solutions, specifically emphasizing values with absolute values of.3 or above derived from a 3-factor solution. FUPC, First Unrotated Principal Component; A, Agreeableness; AO, Adaptive Openness; BT, Braveness-Toughness; C, Conscientiousness; ES, Emotionality/Emotional Stability; EX, Energetic Extraversion; FH, Fair-Minded Honesty; GC, Coal-Oriented Conscientiousness; H, Honesty-Humility; O, Openness/Imagination to Experience (Intellect-Imagination); CA, Combative Aggression; X, Extraversion.

Eigenvalues for the first ten components on the ipsatized data were as follows: 10.8, 8.0, 5.7, 4.0, 3.4, 2.6, 2.2, 2.1, 1.9, and 1.8. We applied PCA to the ipsatized data on the 99 sport team personality traits, followed by Varimax rotation. In addition, all results based on Promax-rotated solutions were very similar. Figure 1 presents the hierarchical emergence of factors from the first unrotated principal component to the six-factor solution. In addition, the figure provided the correlations between the component scores calculated from analyses at each adjacent level.

### One-, two-, three- and four-factor solutions

Regarding the first unrotated principal factor (FUPC), the dimension is primarily characterized by socially desirable vs. undesirable traits. At the FUPC level, the highest loading terms included purposeful, dependable, insightful, organized, confident, self-disciplined, versatile, and optimistic vs. undisciplined, undependable, uneducated, disorganized, unconscientious, unimaginative, uncultured, and insincere. The first unrotated factor closely aligned with the findings observed in previous lexical approach studies to finding personality structure (18, 64). The Disciplined Ingenuity dimension exhibited high loadings for traits representing Conscientiousness (e.g., purposeful, dependable, organized, efficient, self-disciplined) and Openness to Experience (e.g., innovative, insightful, versatile vs. unimaginative, uncultured).

In the two-component solution, the first component, which was the conscientiousness component, remained nearly the same as in the FUPC. The second component of the two-component solution was defined by terms that were interpretable as the Openness to Experience (e.g., introspective, insightful, imaginative, original, inventive, innovative, artistic) dimension as well as positive and negative valence in Honesty-Humility factor (e.g., ethical, respectful, fair-minded, honest, just vs. violent, aggressive, fierce, explosive, ruthless).

In the three-factor solution, the largest factor labeled 3/1 was Conscientiousness. The Conscientiousness dimension remained similar to its representation in the two-factor solution. The second component (2/2) in the two-factor solution was divided into two specific subcomponents (3/2 and 3/3). One of the resulting subcomponents represented a blend of prototypical Extraversion (e.g., active, energetic, enthusiastic, extroverted, lively, passionate, spirited) and Openness to Experience (e.g., introspective, cultured, inventive, imaginative, artistic, innovative, insightful). The third dimension (3/3) was defined by terms indicating Honesty-Humility (e.g., honest, fair-minded, respectful, ethical, genuine, just, vs. boastful) and Agreeableness (e.g., violent, ruthless, demanding, explosive, aggressive, fierce, authoritarian).

In the four-factor solution, Conscientiousness (4/1) was the largest factor of the solution. The second component (3/2) divided into two factors within the four-factor solution. One of the resulting subcomponents was defined by terms such as expressive, lively, enthusiastic, extroverted, energetic, sociable, and exuberant, and was thus interpretable as the Extraversion component (4/4) of the Big Five or HEXACO. The other subcomponent (4/2) from the second component (3/2) was interpretable as Intellect/Imagination, known as Openness to Experience of the Big Five or HEXACO with the highest-loading terms those of imaginative, inventive, innovative, insightful, cultured, introspective, original, artistic, and idealistic. The Honesty-Humility/Agreeableness factor (3/3) in the three-factor solution was the third-largest factor (4/3) of the four-factor solution.

### Five-factor solution

Table 4 lists the highest factor loading terms on each factor from varimax rotations of principal component analysis.

**Table 4:** Highest loading terms on varimax-rotated factors of five-factor solution derived from 99 sport team personality traits.

Factor									
GC		AO		FH/CA		BT/CA		EX	
PTPC traits	Load	PTPC traits	Load	PTPC traits	Load	PTPC traits	Load	PTPC traits	Load
Undisciplined	−.687	Imaginative	−.706	Honest	.667	Nonexplosive	−.577	Lively	.583
Undependable	−.635	Inventive	−.654	Fair-minded	.650	Courageous	.533	Expressive	.563
Disorganized	−.621	Innovative	−.638	Sincere	.638	Brave	.532	Enthusiastic	.546
Self-disciplined	.565	Insightful	−.630	Respectful	.636	Insincere	−.457	Extroverted	.527
Purposeful	.546	Cultured	−.617	Ethical	.626	Fearless	.451	Sociable	.508
Confident	.540	Introspective	−.588	Genuine	.620	Undemanding	−.443	Energetic	.479
Self-confident	.537	Original	−.570	Violent	−.577	Uncommunicative	−.431	Introverted	−.475
Unconscientious	−.516	Artistic	−.546	Ruthless	−.568	Loyal	.426	Spirited	.452
Fearful	−.515	Uncultured	.511	Demanding	−.549	Fierce	.425	Exuberant	.442
Disciplined	.505	Idealistic	−.509	Explosive	−.526	Cool	.302	Vibrant	.403
Uneducated	−.498	Unimaginative	.488	Combative	−.524			Unemotional	−.393
Organized	.488	Masculine	.478	Aggressive	−.490			Excitable	.379
Persistent	.487	Strong	.437	Just	.489			Passionate	.328
Dependable	.462	Tough	.425	Boastful	−.454				
Ambitious	.455	Active	.409	Conscientious	.380				
Decisive	.449	Complex	−.367	Authoritarian	−.377				
Efficient	.417	Assertive	.335	Amiable	.302				
Determined	.411	Self-assured	.334						
Uncalculating	−.407	Versatile	−.316						
Emotional	−.395								
Perfectionistic	.386								
Bold	.385								
Resourceful	.375								
Optimistic	.348								
Resolute	.337								
Dynamic	.333								
% of Variance	7.91	7.07		6.79		5.36		5.03	

Note. Extraction method: Principal Component Analysis. Rotation Method: Varimax with Kaiser Normalization. *N* = 99. GC, Coal-Oriented Conscientiousness; AO, Adaptive Openness to Experience (Intellect-Imagination); FH, Fair-Minded Honesty; CA, Combative Aggression; BT, Braveness-Toughness; EX, Energetic Extraversion.

The four components (i.e., Conscientiousness, Openness to Experience, Honesty-Humility/Agreeableness, Extraversion) of the five-component solution remained basically the same as in the four-component solution. The largest factor of the five-factor solution included self-disciplined, purposeful, confident, organized, persistent, dependable, and perfectionistic on the positive pole and undisciplined, undependable, disorganized, unconscientious, and uneducated on the negative pole.

Therefore, the factor can be identified as the Conscientiousness dimension that resembles the classic Big Five Conscientious factor (46). The highest loading adjectives on the second factor were imaginative, inventive, innovative, insightful, cultured, introspective, original, and artistic on the negative pole. This second factor was interpretable as Openness to Experience of the Big Five or HEXACO. The third largest factor of the five-factor solution included honest, fair-minded, sincere, respectful, ethical, genuine, and just on the positive pole and violent, ruthless, demanding, explosive, combative, and aggressive on the negative pole. This third dimension therefore resembles the Big Five Agreeableness factor, albeit with a stronger representation of Honesty-Humility content of HEXACO.

The fourth factor was defined by high-loading terms such as courageous, brave, fearless, loyal, fierce, and cool on the positive pole and nonexplosive, insincere, undemanding, and uncommunicative on the negative pole. Therefore, the fourth factor can be viewed as a blend of Emotional Stability (e.g., courageous, brave, fearless) and low Agreeableness (e.g., insincere, nonexplosive, undemanding). High loading terms on the fifth factor were lively, expressive, enthusiastic, extroverted, sociable, energetic, spirited, exuberant, vibrant, and excitable on the positive pole and introverted and unemotional on the negative pole. In many respects, this fifth factor resembles the Extraversion factor of the Big Five model or HEXACO. To summarize the above results, the sport team personality five-factor solution contained three factors that could clearly be interpreted as Conscientiousness, Openness to Experience, and Extraversion of the Big Five or HEXACO.

### Six-factor solution

The highest loading terms on each factor of the varimax-rotated six-factor solution are summarized in Table 5.

**Table 5:** Highest loading terms on varimax-rotated factors of Six-factor solution derived from 99 sport personality team traits.

Factor											
GC		AO		FH		EX		CA		BT	
PTPC traits	Load	PTPC traits	Load	PTPC traits	Load	PTPC traits	Load	PTPC traits	Load	PTPC traits	Load
Undisciplined	−.716	Imaginative	.702	Honest	.746	Expressive	.578	Explosive	−.690	Strong	.586
Undependable	−.657	Innovative	.692	Sincere	.741	Lively	.549	Nonexplosive	.631	Courageous	.559
Disorganized	−.640	Inventive	.676	Respectful	.663	Extroverted	.535	Demanding	−.616	Tough	.542
Unconscientious	−.577	Insightful	.633	Genuine	.650	Sociable	.528	Undemanding	.570	Brave	.514
Self-confident	.560	Original	.601	Fair-minded	.600	Enthusiastic	.498	Violent	−.531	Fearless	.480
Purposeful	.555	Introspective	.591	Just	.590	Introverted	−.476	Ruthless	−.528	Energetic	.437
Confident	.549	Cultured	.579	Loyal	.575	Unemotional	−.453	Authoritarian	−.481	Daring	.429
Fearful	−.546	Artistic	.557	Ethical	.566	Spirited	.445	Aggressive	−.475	Assertive	.382
Uneducated	−.539	Unimaginative	−.551	Bold	−.337	Uncommunicative	−.444	Fierce	−.460	Self-assured	.353
Self-disciplined	.529	Uncultured	−.549	Boastful	−.327	Exuberant	.440	Combative	−.446	Cool	.349
Organized	.495	Idealistic	.465	Conscientious	.305	Excitable	.395	Hardworking	.321		
Persistent	.484	Masculine	−.410			Vibrant	.386				
Disciplined	.475	Versatile	.368			Passionate	.373				
Decisive	.464	Adventurous	.365								
Insincere	−.445	Complex	.343								
Uncalculating	−.430	Active	−.335								
Optimistic	.428	Proud	−.315								
Ambitious	.417										
Dependable	.414										
Determined	.412										
Efficient	.408										
Perfectionistic	.391										
Resourceful	.390										
Resolute	.372										
Emotional	−.337										
Dynamic	.316										
% of Variance	8.25	7.06		5.49		4.84		4.65		4.54	

Note. Extraction method: Principal Component Analysis. Rotation Method: Varimax with Kaiser Normalization. *N* = 99. PTPC, Perceived Team Personality Composition; GC, Coal-Oriented Conscientiousness; AO, Adaptive Openness to Experience (Intellect-Imagination); FH, Fair-Minded Honesty; EX, Energetic Extraversion; CA, Combative Aggression; BT, Braveness-Toughness.

The content of three factors (i.e., Conscientiousness, Openness to Experience, Extraversion) from the five-factor solution was nearly identical across the five- and six-factor solutions with correlations above .90 or higher for all three factors (Note that Openness and Experience was reversed in the five-factor solution; hence *r* *=* −.96 with the six-factor version of the factor) (18). In the six-component solution, Conscientiousness (*r* *=* .98), Openness to Experience (*r* *=* −.96), Extraversion (*r* *=* .97) were nearly the same as in the five-component solution. The Honesty-Humility/Agreeableness component of the five-factor solution was divided into two dimensions, such as Honesty-Humility and Agreeableness in the six-factor solution. In addition, the fourth dimension of the five-factor solution has its variance distributed across three components (i.e., Honesty-Humility, Agreeableness, Emotional Stability) of the six-component solution. Furthermore, a new factor from the seven-factor solution includes traits (e.g., passionate, optimistic, resolute, persistent, ambitious, determined, and hardworking). The factor was interpretable as high conscientiousness, high extraversion, and high emotional stability.

### Dimensions of perceived team personality composition

The team personality composition dimensions were derived from the highest-loading adjectives from the six-factor solution. Based on the six-factor solution, this study identifies six key dimensions of team personality composition. First, the Goal-Oriented Conscientiousness (GC) closely aligns with Conscientiousness in the HEXACO model, and reflects a team's organization, work ethic, and commitment to achieving goals (43, 65). Teams high in GC may exhibit structured training regimens, tactical discipline, and relentless pursuit of excellence, key traits that contribute to sustained success in competitive sports. Second, Adaptive Openness (AO) aligns with Openness to Experience, emphasizing imagination, innovation, and strategic adaptability (65). Professional sports teams high in this dimension demonstrate inventive playmaking, creative problem-solving, and a forward-thinking approach to strategy and branding. Teams high in the AO may embrace innovation in tactics, training methods, and fan engagement, which reflects high Openness's exploratory and idea-driven nature in the HEXACO model. Third, Fair-Minded Honesty (FH) aligns with Honesty-Humility, emphasizing sincerity, fairness, and ethical behavior (65). Teams high in this factor foster a culture of trust, loyalty, and sportsmanship, valuing fair play and ethical decision-making. In contrast, teams with low FH may struggle with internal discord, lack of trust, or reputational issues due to boastfulness or a lack of accountability. Fourth, the Energetic Extraversion (EX) dimension corresponds to Extraversion, reflecting enthusiasm, sociability, and emotional expressiveness (65). Professional teams high in EE are known for their charismatic presence, vibrant fan engagement, and strong team chemistry. These teams may thrive in high-energy environments, often displaying dynamic communication and excitement on and off the field. Conversely, teams scoring low in this dimension may lack emotional expressiveness and struggle to generate momentum, leading to a lack of cohesion and fan connection. Fifth, Combative Aggression (CA) may capture a professional sports team's relentless, forceful, and combative nature during competition. This dimension aligns with Geuens et al.'s (13) Aggressiveness (Aggression) in one of the seminar brand personality studies, which includes traits such as bold and aggressive. In this study, CA is characterized by attributes such as explosive, demanding, violent, ruthless, authoritarian, fierce, and combative. CA is often associated with low levels of Agreeableness, of Honesty-Humility, and of Emotionality in personality psychology (65). Sixth, Braveness-Toughness (BT) reflects low Emotionality and high extraversion, capturing a team's fearless, daring, and assertive nature. This dimension aligns with Ruggedness in brand and team personality research (1, 12). High-scoring teams in this dimension may display physical and mental toughness, fearless play, and an assertive presence on the field. In contrast, teams with lower BT may struggle to assert themselves, hesitate in critical moments, or fail to respond aggressively in high-stakes situations, potentially affecting their performance and competitive reputation.

In contrast, teams with lower BT may exhibit hesitation, anxiety, or an inability to handle high-pressure situations, and may result in inconsistent performances.

To establish whether the dimensions of team personality composition represent the HEXACO dimensions well, in Table 6, the correlation between the team personality composition dimensions and the HEXACO dimensions re reported. From the 99 original traits, we identified and retained 85 representative traits based on their conceptual relevance to personality structure. This selection process focused on personality traits previously identified in lexical studies in personality psychology (18, 43, 65). The six HEXACO dimensions were computed using the selected 85 traits, each derived from empirically validated adjectives linked to their respective factors. All team personality composition factors show strong correlations (>.90) with their corresponding HEXACO dimensions, and indicate convergent validity between these dimensions. Although the relationship between reversed Combative Aggression and HEXACO Agreeableness (*r* = .895) indicates a strong association, the highest correlations observed—Goal-Oriented Conscientiousness with Conscientiousness (*r* = .960), Energetic Extraversion and Emotionality (*r* = .956), Fair-Minded Honesty with Honesty-Humility (*r* = .938), Braveness-Toughness with Emotionality (*r* = .935), and Adaptive Openness and Openness to Experience (*r* = .933). The results suggest that these team personality composition dimensions are well-aligned with the dimensions in the HEXACO model. The moderate correlations among different HEXACO dimensions and team personality factors suggest that these constructs are interconnected but distinct, and reinforces the multidimensional nature of team personality composition.

**Table 6:** Means, standard deviations, correlations of perceived team personality factors with HEXACO dimensions.

Team personality dimensions	HEXACO MODEL							
	H	E	X	A	C	O	*M*	*SD*
Fair-Minded Honesty (FH)	.938**	.402**	.411**	.327**	.594**	.456**	5.08	.72
Braveness-Toughness (BT)	.406**	.935**	.825**	−.148**	.728**	.487**	5.93	.86
Energetic Extraversion (EX)	.387**	.608**	.956**	−.104*	.622**	.438**	5.79	.81
Reversed Combative Aggression (CA)	.186**	−.385**	−.359**	.895**	−.219**	−.245**	3.54	.95
Goal-Oriented Conscientiousness (GC)	.470**	.718**	.734**	.011	.960**	.526**	5.64	.79
Adaptive Openness (AO)	.420**	.537**	.608	.000	.693**	.933**	5.03	.83
Mean (*M*)	5.03	5.55	5.90	4.08	5.65	4.69		
Standard deviation (*SD*)	.70	.71	.78	.65	.86	.74		

Note. *P < .05.

** *P* < .01.

## Discussion and implications

### Conceptual, theoretical, and methodological implications

This study provides significant conceptual, theoretical, and methodological insights into sport team personality. Addressing the first research question, this study aims to identify a main structure of team personality traits in the context of sport, employing a lexical approach as the conceptual, theoretical, and methodological framework. The lexical hypothesis in psychology posits that all significant aspects of human personality are encoded in language. This hypothesis underpins the theoretical foundation of the lexical approach, which suggests that the way people describe themselves and others in natural language captures essential personality traits. When applied to sport team personality research, this approach theorizes that significant and commonly observed personality traits applicable to sport teams can be encoded in sport fans' natural language over time. Therefore, the lexical approach, which provides a theoretical basis for obtaining a set of representative personality traits, can serve a theoretical foundation to identify the major structures of sport team personality (30). Hence, the lexical approach, with its theoretical and methodological basis for finding representative team personality traits in sport, served as a valuable foundation for identifying the major dimensions of sport team personality.

Although numerous studies in personality psychology have aimed to include a significant proportion of their scale items as reverse coded or negatively keyed, only a handful of brand personality research efforts have followed this approach or method (39, 49, 65). The inclusion of negatively keyed items in personality evaluations, particularly those based on frameworks such as the Big Five or HEXACO models, is essential for mitigating respondents' acquiescence bias, improving measurement accuracy, and encouraging more thoughtful responses (65). Integrating both positive and negative items in the evaluation of sport team personality traits offers a methodological advancement that addresses the concerns raised by personality psychologists. Consequently, this research has adopted a balanced approach in selecting sport team personality traits to tackle these challenges and underscores the importance of selecting well balanced team personality traits based on the lexical approach.

In addressing the second research question, which explores whether the dimensions of sport team personality are similar to the Big Five or HEXACO structure based on the lexical approach, this study investigated the hierarchical emergence of factors from the one-factor solution to the six-factor solution (46). This study explored the hierarchical structure of sport team personality traits based on factor scores from the six analyses. The hierarchical emergence of factors from the five to six-factor solution derived from ratings on the set of 99 sport team personality traits was highly similar to the Big Five or HEXACO factors. The hierarchical analysis can enhance the understanding of personality dimensions by organizing traits within a structured framework. This approach reveals the intricate relationships among traits and enables the identification of overarching dimensions from individual personality traits (18, 46). The use of hierarchical analysis can be beneficial for identifying dimensions of team personality allowing researchers to systematically organize and categorize the complex and multifaceted traits applicable and relevant to sport teams. The results of this study align with previous findings in sport team personality research with three or four factors within the Big Five or HEXACO.

In the five-factor solution, the space of sport team personality traits contained five dimensions resembling the Big Five. The five-factor solution contained dimensions resembling the Big Five personality factors obtained in lexical studies of personality structure, such as Conscientiousness, Intellect-Imagination-Unconventionality (Openness to Experience), Extraversion, Emotionality, and Agreeableness components.

Furthermore, in the six-factor solution, the Honesty-Humility/Agreeableness component of the five-factor solution was divided into two dimensions, such as Honesty-Humility and Agreeableness. The six-factor solution included dimensions that closely paralleled the HEXACO model, thus confirming the presence of a structure similar to human personality models within the context of sport team personality. This alignment with the HEXACO model underscores the comprehensive nature of the lexical approach in capturing the essence of sport team personality, and demonstrates that teams, like humans, can embody a complex set of traits that resonate with consumers on multiple levels. The division of Honesty-Humility and Agreeableness into separate dimensions suggests a further understanding of sport team personality, where teams can be distinguished not only by their characteristics in the dimension of Agreeableness but also by their respectfulness and sincerity in Honesty-Humility.

### Managerial implications

Our study's findings, which demonstrate the alignment of sport team personality with the Big Five or HEXACO human personality models, provide sport franchises with valuable insights for brand positioning, fan engagement, and targeted marketing strategies. This alignment offers a framework for franchises to deeply understand fan perceptions of their team personality, and enables targeted strategies to enhance market positioning and fan engagement. Particularly for franchises with historical performance challenges, the lexical approach offers a method to reassess and realign their team personality (5). Understanding and aligning their team's personality with fan expectations and values can enhance engagement and loyalty through community involvement and fan experiences rather than relying solely on game results or on-field performances (5).

Regarding sport team differentiation from competitors, employing positive and negative personality traits identified through the lexical approach allows franchises to address weaknesses and highlight strengths against rival teams. Aligning marketing strategies with identified team personality dimensions helps differentiate the team in a competitive market and build a stronger, authentic connection with fans. In addition, regarding enhancing sport team congruity with their sport consumers, the study can examine the congruity between fans' personalities and the sport team's perceived personality when they have similar or exact dimensions (29, 66). Such congruity can significantly influence fan loyalty and behavior, suggesting that sport marketers and managers should craft messages and experiences that align with their target audience's psychological profile.

In addition, although the Honesty-Humility factor is important, most studies rely on the Big Five framework, leading to insufficient exploration of the factor's roles in organizational contexts (67, 68, 73). The measurability of the Honest-Humility factor, revealed in this study, provides sport managers with a valuable opportunity to understand better and leverage this dimension in the brand management of their teams. Organizations can adopt a more diagnostic approach in their strategic marketing efforts, which ensure that their teams project desirable traits while mitigating reputational risks. The sport industry's volatility and social media's rapid influence make teams increasingly vulnerable to sudden and intense public scrutiny. Incorporating the six-factor model to assess team personality in sport provides organizations and managers with a systematic tool to evaluate their brand identity and that of their competitors, strengthening their strategic brand management efforts. This approach may empower them to develop compelling brand management strategies linked to team personality, strengthen stronger sport consumer connections, and foster loyalty and long-term success.

In conclusion, this study advances the understanding of sport team personality by employing a lexical approach to identify its major dimensions and examining their alignment with established human personality models. The findings reinforce the applicability of the Big Five and HEXACO frameworks in the sport context by offering valuable insights for researchers and practitioners. By systematically analyzing the hierarchical emergence of personality dimensions, this study provides a structured foundation for future research on team personality composition. Furthermore, the strategic implications of aligning marketing and branding efforts with identified team personality traits reinforces the practical relevance of this research. In addition, given the limited exploration of the Honesty-Humility factor in sport organizations when relying on Big Five models, this study highlights the dimension's potential significance in sport brand management. Ultimately, this study contributes to the growing body of knowledge on sport team personality and its impact on organizational differentiation and consumer connections in the highly competitive sport industry.

## Limitations and future research

### Limitations

There were a few limitations to this study. First, it is essential to recognize that fans' interpretations of a team's personality may not remain static. The data collected in this study reflected fans' perceptions of certain organizations at a specific point in time. However, fans may perceive a team's personality differently during competition than in non-competitive settings (i.e., community service appearances or media interactions). Additionally, a team's perceived personality may evolve over time.

Second, the regional or national culture of sport where participants lived could impact how they perceive the personality of teams or athletes in their nation. Furthermore, the study included only 99 personality traits both applicable and relevant to sport teams from 499 potential personality traits selected. Future research should examine larger variable sets that may approximate the entire sport team personality domain.

Third, this study did not conduct comparative analyses across teams due to limitations in sample size and response distribution. While comparative research could offer valuable insights into whether certain franchises exhibit distinct personality profiles, the unequal number of respondents per team and potential response biases in the dataset posed challenges for reliable statistical comparisons. Future studies with larger, more balanced, and representative samples could address these issues and provide deeper insights into how team personality influences fan perceptions, engagement, and loyalty.

### Future research

Given the present study's focus on the perceived team personality of a sport fan's favorite team, one area of future research is conducting surveys with fans about other sport organizations they are familiar with but do not consider their favorite team. For instance, researchers could examine how fans perceive the team personality of their favorite team's rival in college and/or professional sport. For example, previous research suggests that fans perceive themselves as changing how they consume sport based on whether their favorite team competes against a primary or secondary rival (69, 70), therefore, fans could also hold different perceptions about the personality of rival teams compared to their favorite team.

Researchers could also build on previous work related to the personality of sport leagues or competitions. A previous study examined brand personality of the NFL (30), so other studies could focus on how fans perceive certain professional or collegiate leagues as having personality attributes. Whether there is a relationship between fan perceptions of personality attributes and demographic factors, such as age, gender or race, or behavioral factors, could also be investigated. For example, female sport fans have been found to have unique reasons for consuming sport compared to males, so personality attributes could vary by gender (71). Behavioral factors, such as how often a fan attends or views broadcasts of a game, might also influence how they perceive personality attributes. Finally, the present study investigated how fans perceived the personality attributes of their favorite team at one point in time. A longitudinal study could examine how fan perceptions of a sport organization's personality attributes change over time. Certain factors, such as a fan aging or having new social experiences, could cause the perceptions of personality attributes by fans to change (72).

Market research, including social media analysis, can provide timely information and identify trends in consumer behavior. Customer feedback surveys and online reviews provide insight into what attracts and detracts consumers to specific goods and services and to which providers. This research should be conducted on a regular basis to stay connected to the pulse of the market, and allow for early detection of a change in consumer interactions or development of a new trend. Once market research has been conducted to gain a general understanding of consumer interaction trends with key competitors, the personality attributes of those competitors should be analyzed. An analysis of personality attributes within the competitive landscape of a sport organization allows sport managers to identify high indications of personality factors and accompanying traits of successful organizations and strategies (6).

In addition, organizations should evaluate themselves along with their competition. For example, if an analysis of a successful organization indicated a high Openness to Experience factor, that organization would show traits of being imaginative, innovative, inventive, and insightful. This would be identifiable in their marketing strategies, customer reviews, and interactions with their employees. The organization using this method of analysis could then schematize to recruit employees and acquire strategies of their own with these traits along with establishing development programs that would strengthen and project these traits. The execution of such a reflective and panoramic evaluation will provide valuable insight to specifically targeted consumer interactions, preferences, loyalty, and prioritization.

Furthermore, regarding the relationship between the Honesty-Humility factor and the aforementioned predicted variables in personality psychology, exploring the conceptualization of the Honesty-Humility factor in the context of sport brand management may offer a more valid explanation for understanding sport consumption behavior. For example, future research can explore the relationship between the Honesty-Humility factor of athlete endorsers as human brands and several predicted variables (e.g., athlete-endorsed brand image, brand association with the athlete, consumer's brand attitude).

## Data Availability

The original contributions presented in the study are included in the article/Supplementary Material, further inquiries can be directed to the corresponding author.
